# Self-Narrative in a Therapeutic Group Pathway for Cancer Patients: Discussion of the Group Narrative Psychotherapy Intervention Initiated at the Veneto Institute of Oncology: IOV IRCCS of Padua

**DOI:** 10.3390/bs14050376

**Published:** 2024-04-29

**Authors:** Letizia Iannopollo, Grazia Cristaldi, Alessandra Feltrin

**Affiliations:** 1 Hospital Psychology, Veneto Institute of Oncology IOV-IRCCS, 35128 Padua, Italy; alessandra.feltrin@iov.veneto.it; 2 Freelance Psychologist, 35128 Padua, Italy; cristaldigra@gmail.com

**Keywords:** empowerment, compassion, quality of life, traumatization, awareness, distress, anxiety, coping, pain, narrative medicine

## Abstract

The following article proposes a reflection on the experience of Narrative Therapy of a group of cancer patients, non-homogeneous for sites and stages of disease, participating to a therapeutic writing path, in order to process the trauma reactive to cancer and to reflect on themselves. Taking inspiration from the assumption that “writing helps when people are faced with a stumble”, facilitating the process of distancing from life-events, each writer establishes either context, in a more intimate and true way, or memories and emotions connected to it, in order to express them to the group and to themselves too, and to identify new adaptation styles. The therapeutic path lasted eight meetings, during which some themes were chosen to guide the written reflection, which was carried out at home, and then shared with the group. The therapeutic path is an opportunity to adjust the perspective with which the specialist accompanies patients during the adaptation process to the disease, moving from the “explanation” to “comprehension”; from symptom to “sense”. The group narration is based on a relational perspective of co-construction of the meaning of experiences, highlighting the different roles and relevance of the factors involved.

## 1. Introduction: History of Narrative Therapy

In the last few years, the possibility of cancer survival has become increasingly greater, especially thanks to prevention programs, early diagnosis and treatment innovations. Despite this, the diagnosis of cancer and the experience of illness remain highly traumatic [1], with multiple and deep effects on the patient’s life [2]. Focusing on psychological critical issues, there are some risk factors in the adaptation process, such as intrusive thoughts (for example those inherent to the theme of living and dying, the loss of role and body changes) and avoidance (relating to thoughts and feelings, as well as places and people connected to traumatic events) [3]. These two components may derive from traumatic experiences that can evolve into pathological states (such as post-traumatic stress disorder), characterized by emotional (anxiety and depression) and psychosomatic (pain, fatigue, sleep disturbances) symptoms, as well as a reduced ability of adaptation to new living conditions [4].

The field of evidence based medicine (EBM) is not enough on its own to achieve the best possible “global care” outcome. There are many studies that confirm how, alongside oncological treatments, it is necessary to support patients and family members and improve their quality of life (QoL) [5]. In this direction, “the patients’ narration of their own experience allows them to identify and implement coping strategies (adaptation to events)” [6].

Narrative methodologies allow the expression and analysis of personal experiences in the oncological context, encouraging, both in patients and doctors, the organization of thoughts and hopes, the identification of problems, the communication of information, the exploration of possible choices, distancing from the situation, and the reconsideration of personal values [7].

Furthermore, through narration it is possible to strengthen the therapeutic alliance in order to identify more effective treatment paths. The narrative of patients and healthcare workers must be included within the global therapeutic process of care, supporting the patient’s active participation in therapeutic choices, as well as the communication and mutual support (empowerment) of all the parties involved: the multidisciplinary team, the patient and their family [8,9].

Evidence based narrative medicine (NEBM) is considered one of the possible strategies to optimize the approach to the patient as a whole, considering the human being rather than the organ or symptoms. The use of specific skills such as active listening, empathy, and compassion are fundamental to understanding the illness experience, allowing the specialist to establish a functional relationship for the act of care [10,11].

Narrative medicine is based on a willingness to “listen to the patient’s personal life”, which provides an opportunity for the psychologist to build a bridge between distant worlds, with the aim of writing a story that integrates the personal history and the narrative of the doctor. This narrative is co-constructed, intertwining scientific data and adapting them to the experience of the person and their family members [12].

Therapeutic writing meetings can reveal how the person who narrates does not just give information about himself: he shares how he sees the world, how he comprehends reality, how he places himself towards others. In other words, the listener discovers the patient’s language, the meaning he gives to words, concepts and metaphors, and finally witnesses the emotions that the narrator feels.

Therefore, the act of writing means relating to oneself and others, but it also means discovering one’s uniqueness and beauty. From this perspective, the ability to listen is as important as the ability to tell the story [13].

### Theoretical Reference Models

Medical Humanities [14] are a set of humanistic, social and artistic disciplines, placed at the service of medical practice. Considering that health is not only related to physical well-being but also to a psychological and social state, medical humanities integrate scientific knowledge of the body with humanistic knowledge of the patient’s experiences, within his/her personal history. Psycho-oncology promotes medical humanities by offering to those who live the experience of oncological disease, new opportunities and tools for personal growth, integrated with conventional and psychotherapeutic oncological treatments [15].

The theoretical models of narrative medicine are very ancient and find their origins in the work of James Penne-Baker [16], the promoter of “expressive writing”: a psychosocial intervention useful for studying the psychological and psychophysiological mechanisms that they manifest, putting traumas and stressful experiences on paper. Writing about traumatic experiences can generate positive effects on health, especially by modifying the emotions related to a stressful event and re-elaborating the emotional distress related to it. In this way it is possible to recover one’s identity and, at the same time, improve coping skills [17].

Expressive writing is based on basic regulatory processes such as attention, habituation and cognitive restructuring, which reduce the impact of intrusive thoughts on a person’s mood and quality of life. These processes also reduce arousal linked to stressful thoughts and feelings. In fact, it is true that writing about a traumatic event, such as an oncological disease, even if it is accompanied by memories and intrusive thoughts, facilitates the processing of those contents of “avoidance” and anxiety, which usually lead victims to distance themselves from stimuli that could renew the memory of the painful experience. The cognitive reprocessing of the traumatic event allows them to associate a new meaning to the event and insert it into a new and more coherent narrative of themselves and their life [18].

A second theoretical reference model is written exposure therapy (WET), which takes its origins in studies conducted on emotional processing and the extinction of fears. According to experiments, trauma survivors experience significant relief by writing about their traumatic experience [19]. Another distinctive feature of this model is resilience, a psychological construct that can be learned by promoting reappraisal and reframing of the traumatic experience. Thanks to writing-based therapeutic programs, patients learn that traumatic memories are not necessarily dangerous, that the stress associated with memories is transitory and decreases over time, and that physiological responses (such as increased heart rate) are not dangerous, and that it is possible to give new meaning to the traumatic event [18].

## 2. Effects of Trauma on Self-Narration: How the Narrative Changes following Trauma

Reflecting on the experience of oncological disease and the trauma connected to it, it is necessary to consider the impact that the diagnosis has on the person and their family members; in every area of existence (family, work and social relationships) [5] the existential path undergoes a sudden and drastic interruption [19]. Concerns, suspicions, even imagined hypotheses are unable to express the dramatic and shocking experience that a subject faces with the communication of a diagnosis of cancer. In this regard, Bessel van der Kolk states: “New knowledge on the survival instinct explains why traumatized people experience intolerable anxiety and anger, and allows us to understand the impact of trauma on the ability to concentrate and memorize, and on the possibility of establishing relationships based on trust, feeling “at home” in one’s own body” [19].

In the common imagination and in modern Western culture, the concept of health is the antithesis of that of oncological diagnosis; being healthy or having cancer are constructs that cannot coexist: this is observable when people witness the communication of an illness diagnosis. The identity that had accompanied the person up to that moment (influencing experiences, choices, relationships, etc.), suddenly shatters. The experience that patients and their family members usually describe is of feeling muffled, and distant from the room they are in [19]. In clinical practice, professionals witness on a daily basis the experience of patient self-fragmentation as a self-defense mechanism from an emotionally unsustainable situation [20]. Mental confusion appears to be a consequence of a complex psycho-emotional experience in which the patient perceives himself as fragile, helpless, and wounded; emotional and sensorial activation undergo an upward peak, while the ability to think and therefore to regulate the experience through an analysis of reality, requires a different time, necessary to carry out complex analytic processes without the support necessary for an adequate relationship with living reality.

This can lead to people being unable to access their own internal resources to relate to reality, while they re-activate some reactions to events which are conditioned by the instinctive survival reactions. In difficult situations, the organism uses ancient strategies that are already known and that were used in response to past difficult events in which the person who experienced himself as defenseless and with no way out managed to survive [19].

**Figure 1:**
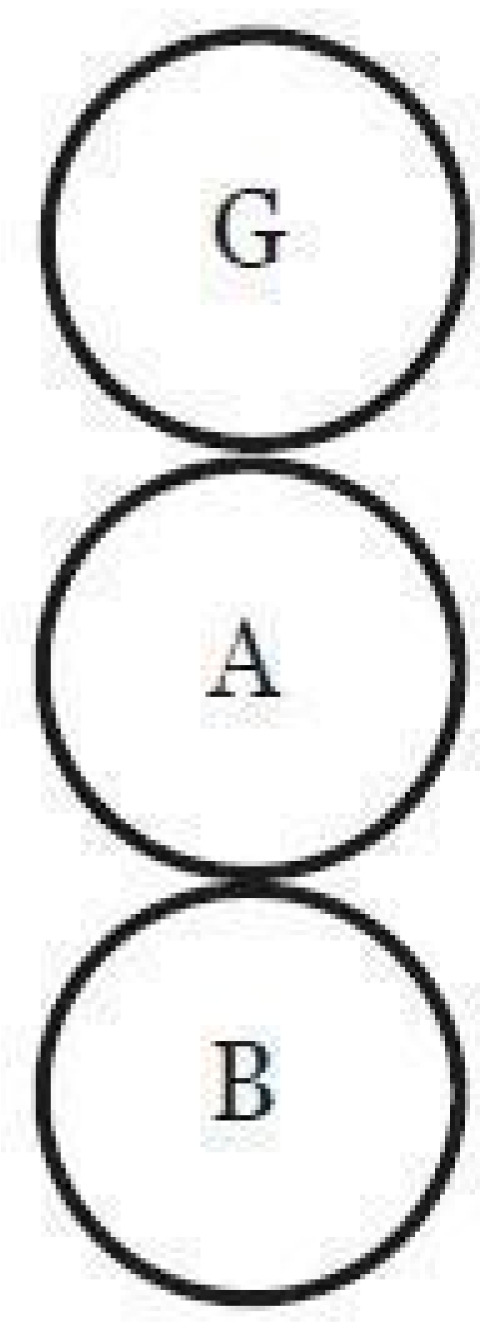
The ego states.

This type of reaction to trauma can be analyzed using the “transactional theory paradigm” [21]. This theoretical approach analyzes the components of everyone’s personality, distinguishing within it, a child (B), an adult (A) and a parental part (G) (Figure 1).

**Figure 2:**
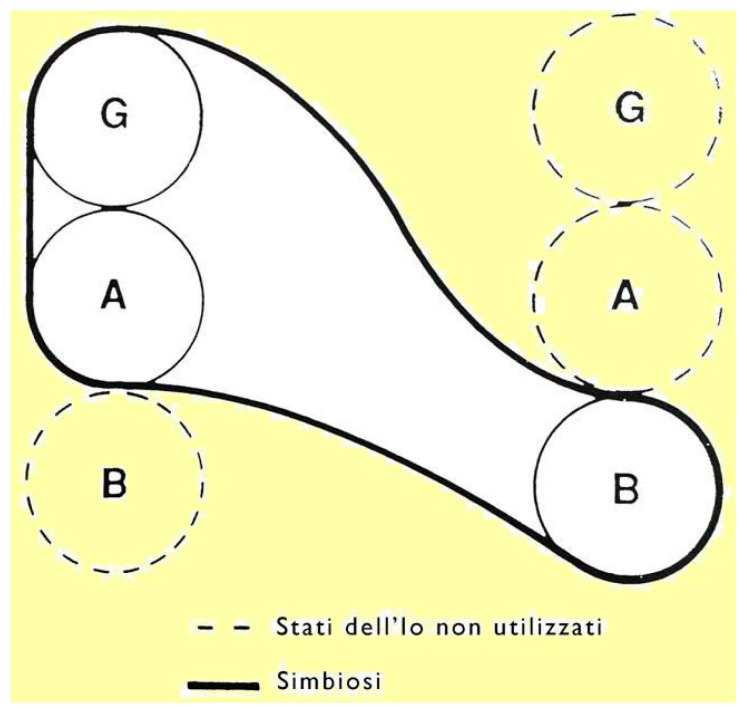
Emotional disorders in the light of Jacqui Lee Schiff’s model.

Transposing this theoretical model to the oncological context, the doctor–patient communication inevitably places the latter in an inferior position, in terms of knowledge and competence, but above all, due to the dependence that the person has on the doctor, who is seen as a “savior”. The result is an encounter characterized by transactions conditioned by the “up” role of the healthcare personnel and the “down” role of the patient, in which the healthcare worker provides the tools, and guides those who find themselves in a hostile and unknown context, offering the “treatment” instructions. Patients are instinctively and emotionally led by the experience of illness to act, being the weak subjects, without competence and lacking in knowledge, experiencing themselves as incapable of thinking, feeling and behaving as adults, predisposed to depend on the specialist, like children (B) with their parents. This involves a physiological regression, which is witnessed during many conversations, which leads adults, who have temporarily lost their professional, social and familial identity, to start symbiotic relationships; the patient uses knowledge and already verified reaction patterns to either protect himself from danger or obtain protection from the healthcare personnel (Figure 2).

These relational structures (transactional) unconsciously influence the way in which the patient deals with the experience of the illness, influencing certain types of experiences, thoughts, actions and choices; a person, who lives an experience as a “child” will inevitably relate to all the aspects that it entails, as well as how he would have reacted in a situation where his evaluation of reality was less aware than that developed in adulthood. A mature subject has self-knowledge based on experience, has defined his own reference values and the tools to understand phenomena and life itself. As a result, he has acquired greater freedom of choice and achieved a certain self-esteem and identity [22,23].

Using a transactional analytical perspective of the doctor–patient relationship, it appears useful to know that the first meetings are interpreted as “the patient’s physiological regressive moment in the face of a threat” [24]; the reactions can be evaluated as requests for help and fear; they can change, evolve, with an appropriate elaboration of what is happening to the patient and to the family. It thus becomes crucial to give patients enough time to recover an adult position, so that they can be aware of their emotional state, as well as of their defenses to face a trauma that would not otherwise be possible to face [25].

### Narrative Focus

According to narrative therapy specialists, it appears clinically relevant to provide a space for processing the traumatic experience and the coping process of the patient who faces his specific illness experience.

An effect of suffering is also the tendency to isolate oneself [25], which is also a consequence of an alteration in self-esteem. This side effect of the illness experience leads to the risk of not allowing the patient to recover his dimension of “person”, and his specific way of being in the world, running the risk of considering needs and future plans that are only related to the disease, with a progressive deterioration in the current and future quality of life.

The elaboration process is stimulated and implemented primarily by the presence of a group [23]; “a safe base in which the person can feel welcomed and which can afford him to trust himself and other people: through the experience of re-understanding reality, the patient can become aware of himself again, welcoming and forgiving his own limitations, fragilities, humanity and personal value” [26].

Through group narration which gives the opportunity to share psycho-emotional experiences and meanings, people experience the feeling of appearing fragile, they also receive a message of acceptance, which allows them to redefine their identity and self-esteem, functionally integrating the traumatic experience with a conscious plan of life.

Unlike the relationships between child and parents, the doctor–patient (D–P) relationship does not allow the patient to become a conscious actor of his own choice; the patient and his family members do not acquire adequate medical skills, nor can they independently choose the most suitable treatment procedure. They can learn to trust the medical team, entrusting their hopes of treatment to others.

The objective of psychological rehabilitation in oncology is to start a therapeutic path that allows the patient to acquire awareness and a functional perspective on life and treatment, to face, process, and accept the change that the illness event has brought about [27].

The energy and effort put in place by the patient and his family should not be consumed by taking responsibility for the medical aspect of the treatment. Therefore, it is necessary to develop the ability to understand how this experience can be addressed, respecting everyone’s needs, limits and resources.

To support the elaboration process, which allows the patient to recover the adult position (A), it can be useful to encourage the patient’s ability to “think” about himself, the disease, treatments and the upheavals faced from the diagnosis [28]. According to our clinical experience, these instruments can help the patients during the healing process, as well as with the psychological support mediated by narrative medicine, which can give them the opportunity to rebuild a subjective and unique meaning for themselves, their family and social support network, and for their social role to be functionally integrated into the illness experience [29].

American experiences [30] show how the comparison of narratives between peers, through self- and mutual-help groups, provides the possibility of starting to establish equal relationships with others, between adults (A–A).

Therapeutic groups [31] allow, in addition to therapeutic work, also freer relationships, in which each person can discuss, reflect and bring into play parts of themselves that cannot otherwise be used, due to the high frequency of hospital environments. The comparison with the group allows the possibility of recovering the memory of themselves in a global sense, to be included in the processing work; they can begin to reconnect the threads of memory, remembering how they were before the trauma. They will be able to access different parts of themselves, to experience trust in a context in which they find themselves with the security of knowing they are protected, welcomed and loved for who they are [32].

The group setting allows people to feel part of a safe, intimate, restricted social context, in which they are stimulated and relate to others as people (human beings), as well as patients (patient); with the succession of meetings and proposed themes, participants are guided to establish a relationship with themselves and with others characterized by trust, respect, welcome, non-judgment, and acceptance. This environment allows everyone to work on their defensive structural reaction to trauma, connecting to parts of themselves that were isolated by suffering, recovering the memory of their previous identity which the illness has interrupted, changing the perspective and the very meaning of how they are in the world. This leads participants to recognize (re-think) what the illness and trauma have determined in themselves. Some examples of possible reactions are: increased activation of alert mechanisms, tightening of defense mechanisms, and protecting the physical and psychological self through the tightening of thoughts, emotions and behaviors, with the aim of not feeling suffering. However, these reactions have the side effect of not allowing access to the fundamental resources and skills to protect one’s psycho-social well-being (such as the historically constructed sense of self, future perspective and hope), effectively relegating the person to an experience of a patient who is committed to following a treatment process, losing awareness of a global self, of his present situation and of the reality that surrounds him [33,34].

The avoidance of pain, trying “not to feel”, involves the interruption of every natural process of processing suffering, thus preventing understanding, acceptance and ultimately overcoming the experience itself [20]. 

Overcoming trauma implies the ability to proceed with the maturation process through the event itself; overcoming doesn’t mean forgetting. Studies on trauma, whether sudden or repeated during a period of life, have now well established the evidence of how the memory of events is not erased from our experience [35]. Non-conscious events and consequences remain as part of our memory, conditioning the other experiences; they continue to give their influence, albeit unconsciously through automatic patterns used on a daily basis. Someone who has lived through a traumatic experience may not have a clear and lucid memory of the event but, nonetheless, may act in a manner that is influenced and conditioned by the effect that it has had, and that it continues to have on their personality organization, defenses, behaviors and the quality of their thoughts and emotions [34,35].

Consequently, the idea of oneself automatically changes, as well as one’s consideration of other people and one’s future expectations, as they are based on one’s ability to process and integrate the traumatic experience into the overall life experience [35].

## 3. The Concept of Automatic Schema and Script in the Experience of Processing the Illness

During therapeutic writing meetings, patients frequently become aware of their own past narrative, which represents a recurring modality for giving meaning to their entire life path and identity as children, as spouses, as workers, etc., even if they were not conscious of them previously.

After the initial shock phase of diagnosis [20], each participant restarts his existence and gives a rhythm to daily life, which now also includes visits, treatments, tests, etc. During the therapeutic writing process, patients begin to recognize ways to overcome the difficulties that are reactivated, giving a bitter sense of continuity to the idea they have of themselves (it is common for beliefs such as: “I’m always the same, bad things all happen to me, I have to be strong, I’m a warrior, I can’t do it alone, I’ll do like my mother, no one understands me, etc.”) [36].

The group context first of all allows them to notice these mechanisms, through the mirroring of others, comparison, discussion, and acceptance of the beliefs expressed. Finding certain mechanisms and ideas as universal allows people to observe them in others and relate them to reality, “re-processing” it. This happens because, when they look at the other from a different perspective, since it is another person’s experience, they can be more rational and understanding, free from fears regarding their personal experience. In this way, they can therefore verify how arbitrary an idea is and how other people experience illness, while they give themselves permission to imagine new possibilities [31,37].

The new relationship with a person, within a therapeutic writing group, allows people to use a different relational (transactional) position; long after the diagnosis, we can observe the illness event and the suffering of other people, leaving the symbiosis and using the adult (A) position, as well as the parental one, and activating the skills and values that belong to them which allow us to act in mature ways [28,29].

## 4. Communication of Diagnosis, from Explanation to Listening: The Different Narratives Used by Patients, Healthcare Workers and Caregivers

The multiplicity of psychological interventions that are applied during the clinical path can be summarized with the words: support, coping skills and psycho-spiritual therapy. Each of these concepts summarizes different problems, phases of the disease and therapeutic approaches, both in terms of intervention methods and objectives to be achieved. These three concepts summarize an intense and painful clinical and human temporal journey, telling the complex existential journey that is summed up by the phrase “from the symptom to the search for meaning” [37].

In the relational experience that occurs when a diagnosis is communicated or received, some factors such as knowledge, expectations, perspective and objectives come into play; as well as personal sensitivity, communication style, competence and ability to take an observational perspective, and to adapt the content making it understandable to others [37].

When a professional communicates a diagnosis he must have a self/hetero observation method [38], to monitor the different levels and important factors that characterize the experience of both the person communicating and the person receiving the “bad news”. It is not enough to assume that the interaction takes place between consenting and capable adults, it is the specialist’s task to remember that the care relationship at the time of diagnosis is characterized by an asymmetry. His role requires the attention, support and care towards those he meets, who are in a relational position of weakness, and who may attribute greater power and knowledge to him and consequently trust him, as a human being, at least initially, attributing salvific value to his service. Consequentially the following levels should always be considered:

- the drama, the news of illness;
- communication of the diagnosis;
- listening to the diagnosis;
- the presence of the caregiver;
- the hospital context in which the news is given;
- the quality of verbal and non-verbal communication;
- the compulsive search for information found on the internet;
- the presence of other healthcare workers and their communication;
- culture, age, social role and future expectations of the entire family;
- the history of other mournful events in the family life;
- beliefs about himself and his life plan.

During medical visits, professionals usually witness the meeting of two different worlds, two different languages and two different objectives. Meeting after meeting it is important to find a common language, open up to others, get to know each other, and build a language that allows a relationship of trust, communicating information about the disease and treatment, and above all to encourage the sharing of realistic and achievable objectives, allowing the identification of effective strategies to recover adequate psycho-emotional and social well-being [39].

In the context of care, we are faced with the necessary consideration and awareness of the contractual aspects that characterize a patient’s informed consent, which the specialist must learn to determine by establishing his method of approach to those in front of him; often a patient gives consent when he may not understand the information received; faced with the fear of pain and death, he cannot help but share a proposal that is often based on desperation, on experiences reactive to a moment in which the trauma caused a shock reaction. In this phase of the treatment relationship the specialist must remember that the therapeutic alliance will be built during the time of cure [39].

The presence of a multidisciplinary team allows the specialist to discuss the case, compare and reflect on the identified needs, resources, characteristics of the patient and caregivers, in order to activate therapeutic choices that can make the treatment project more effective. From a medical point of view, he will have the most innovative evidence-based choices, while from a human point of view he must ask himself “what the subject needs” to find an adequate psycho-emotional and social balance to assume responsibility and therefore choose to activate their treatment path. This allows the patient and his group to activate the resources necessary to protect the very meaning of the experience he is having [40]; the specialist can indicate the activation of psychological support, sharing groups, an external support network to hospitals, etc.

## 5. Therapeutic Effect of Telling Oneself

The literature [41] finds that writing allows people to deal with previously inhibited emotions, metabolize and reorganize traumatic events and memories, generating more adaptable and acceptable internal patterns.

Furthermore, a positive effect of writing about a stressful event is the interruption of the flow of negative thoughts which, among other things, also lower the immune defenses [40].

Psychotherapies that use autobiographical writing techniques are defined as “talking cures”, or word therapies; they use narrative as their focal point. In this context, the story takes on a new overall vision in which both the narrator and the listener are actively understood. History is at the center of the healing process and allows the patient to build a more participatory, shareable, and understandable theory of himself and the world [41].

Autobiographical writing can represent the first step in taking care of oneself and freeing oneself from internal constraints, entrusting one’s experiences to a blank sheet of paper. The creative process that includes remembering and giving people time to write and looking inward becomes therapeutic. Taking care of oneself through the sharing process becomes a process of internal maturation, an act of individual freedom, with the aim of facing the fear of illness and death [42].

In the field of modern oncology there is widespread recognition of rehabilitation as an integral part of the patient’s treatment plan. Together with surgery, chemotherapy and radiotherapy, rehabilitation must intervene in all phases of the diagnostic/therapeutic process, with the aim of maintaining and improving the quality of life [35]. The rehabilitation program must be diversified, multidisciplinary, personalized, flexible, and also established on the basis of the patient’s psychological habitus, as well as the psycho-social context in which he lives [15].

The overall care of the cancer patient must necessarily include a new culture of rehabilitation, understood as the right to have the best possible quality of life, at any stage of the disease, with a consolidation of networking at all levels. Therapeutic writing, reconciling the knowledge of the literary sciences with psychological ones, represents a valid medical humanities intervention for cancer patients from the perspective of the integrated psycho-oncological approach (API) [15].

## 6. Therapeutic Objectives of Narrative Therapy in Oncology

The objectives of narrative therapy represent the structure and essential container of the process of self-narration within the therapy group:

- the creation of a safe setting,
- the creation of an equal social network,
- the narrative context focused on specific shared events,
- the encounter with the self through the use of a blank sheet of paper; the story, the experiences and the meanings chosen to narrate,
- group sharing, attunement and mirroring; the ego that becomes us and returns a more complete and complex self, from the idealized to the real,
- the final farewell and the closing rite to return to a personal life path, transformed by the experience: what I want to leave and what I want to take with me.

## 7. Project Analysis

### 7.1. The Group Journey

On the basis of the theoretical and practical evidence explained so far, the clinical and experimental team belonging to the U.O.S.D. of Hospital Psychology of the Veneto Institute of Oncology (IOV), I.R.C.C.S of Padua, has theorized interventions that could introduce, during the different phases of oncological treatment, a space for group processing, through the technique of therapeutic writing, favoring the simultaneous and gradual de-hospitalization of the daily activities that engage the patients and caregivers during oncological treatments. To make this transition more fluid and natural, a space was chosen inside the Institute, which “symbolically” marks the transition from being a patient to being a person, allowing moments in which to return, little by little, to meet other people with whom to rethink themselves and reinvent themselves. The group journey lasts six months for each edition; the goal is to enable participants to:

- find a temporal dimension (past and present),
- recover the aspects that characterize personal identity, resources, and fragilities,
- reconsider their own experience of illness, which can be thought about, understood, processed and accepted, at their own pace and ability.

Recognizing the aspects of similarity and those of differentiation between oneself and the other, allows one to “recognize” oneself meeting after meeting, writing after writing, alternating moments in which one becomes capable of thinking alone with others, in which one reflects and shares with the others. It is a delicate and intimate journey in which the person is accompanied, protected and stimulated to regain the confidence to observe the experience with their own eyes, mind and senses: returning to believe in their ability, to understand the experience they are experiencing [43].

Recovering self-esteem in this situation requires the ability to evaluate the entire experience, understanding the difficulties and pain faced, the information received and the support concretely offered. Patients can then better understand their needs and fears, as well as verify the strategies used to deal with them and identify new ones, which reflect the new coping methods used [44,45].

The last step involves thinking about future planning: what to expect, what might be needed and how to act to satisfy these needs.

### 7.2. Therapeutic Objectives of the Project

The following therapeutic objectives were considered for the entire group and the individual participants [23].

1. Allow the emergence of internal mechanisms of adaptation to the experience, in a path of awareness, processing and recognition of the narrated self-image. In this way it is possible to “update” one’s beliefs and interpretations of reality, subsequently identifying unused internal resources. To do this it is useful to build a story structured by some specific themes.
2. Rediscover the value of sharing and assume a “meta-group” observation perspective, with which the participants are able to see themselves, through a different, alternative, flexible, human interpretative lens. This is made possible by the rediscovery of values and principles, shared by the group, which favor an evolution of the relationship with one’s internal objects, which can be guided by realistic principles, based on current needs, for care and understanding and abandoning more self-centered ego positions.
3. The last step of the group journey consists in thinking about the future: what could be the appropriate planning based on the treatment process, the personal and family projects that can be pursued, the feasible work possibilities. The future represents the desire to respect oneself and the identity and existential aspects that have emerged through the narrative path, which provide the person with a new self-awareness.

## 8. Data and Results

The narrative therapy process welcomed 36 women, in different stages of illness and treatment.

At the beginning of the process, the patients shared beliefs and convictions about themselves, about others and about the disease, with a prevalence of negative connotations: low self-esteem and a limited sense of efficacy, disorientation and uncertainty regarding future prospects, i.e., issues that are emotional, sexual and work-related. Moderate emotional dysregulation, which led patients to focus on sharing moments of fear, anger, suspiciousness, and frustration. At the same time, a decrease in the ability to socialize, an increase in the experience of isolation and a reduced ability to enjoy the social interests carried out before the illness were noted [44]. From the participants’ sharing, a strong need for discussion with medical and healthcare personnel was revealed, which would allow them to understand, evaluate and plan their own therapeutic path.

A negative connotation was revealed in the description of relationships with family members and with the social context: in many testimonies the patients did not feel adequately listened to and understood, reporting that the presence of others was more useful for protecting and containing the anxieties and concerns of those who cared for them, rather than for themselves.

The therapeutic process allowed the participants to express and work on needs such as: better communication; finding moments of true sharing and intimacy with family and friends; the possibility of having time, after the diagnosis, to put the change started into practice, through active choices; to understand what changes (physical and psychological) they will face in the future; the desire to be considered normal people.

## 9. Conclusions

The narrative psychotherapy experience took place at the U.O.S.D. of Hospital Psychology of the Veneto Institute of Oncology (IOV), I.R.C.C.S of Padua, in the period from 2021 to 2023. It involved 36 cancer patients followed at the Institute, with different diagnoses and prognoses, some of whom were at the stage of advanced metastatic disease. The initial aim was to start a group psychotherapy process that would corroborate our clinical evidence with the introduction of an additional phase in the psycho-oncological treatment process, giving an appropriate space to group psychotherapy, to difference the treatment from how it is currently offered to patients and their families during the actual oncological treatment process.

**-**  **Limitations of this work:**

*We would like to inform readers that the article aims to highlight a methodological choice of care, reporting the experience of the working group, but does not carry out a systematic review of the existing bibliography nor does it expose clinical research data.*

The article continues with the aim of drawing the attention of the scientific community to the role that could be played by systematically introducing into psychological management the practice of starting a path of personal psychological support (evaluated in a multidisciplinary team), and a subsequent phase of treatment through group psychotherapy. According to our clinical experience, the transition to the group would allow us to review the objectives achieved in the personal journey, encouraging psychoeducation, sharing and strengthening survival strategies through a group vision. The benefits of the presence of a group with which to find relationships on an equal footing have already been considered previously. The same effective and supportive dynamics of the group have the function of providing the subject with the possibility of experimenting with the mechanisms of sharing and of mirroring one another and of feeling welcomed and accepted by the group. In group psychotherapy they can represent a fundamental rebirth factor to support the process of acquiring a new meaning of oneself.

**-**  **Initial considerations:**

Currently, according to previous data in the literature, psychological support for cancer patients is structured through a process of taking charge which follow a request, from the patient or the oncologist, of supporting the patient and his family during the recovery process care, in order to promote compliance and adaptation and to maintain an adequate quality of life during and after treatments.

This type of the patient’s psychological care generally occurs in a phase of illness, in which the subject expresses psycho-emotional distress, which compromises adherence to therapies or his psychological balance. This suffering can lead to psychological or psychiatric symptoms that significantly impact quality of life and psychological well-being. The psychologist therefore intervenes following the detection of the discomfort, when it is already being expressed due to the repercussions of the ongoing treatment process. The objective of psychological support is to re-establish sufficient emotional compensation and trust in the ongoing treatment process.

**-**  **Therapeutic hypothesis:**

Since 2019 we have introduced the group therapeutic activity based on narrative therapy, written and oral, among participants who share the same state of oncological disease. The patients, 90% of whom were women, with different locations and phases of the disease, had largely already followed a path of personal psychotherapy, to support the difficulties connected with the trauma experienced, as well as the disease, the side effects of the therapies, the interruption of the previous life plan, the uncertainty about their socio-work future, and personal identity. Therefore, they were enrolled for a group therapeutic journey, agreeing with the idea of starting a journey within themselves, first recognizing and talking about themselves, in their illness, in their suffering, and then finding themselves in their past, in their memories and projecting themselves in the future, feared or hoped for [14].

The participants experimented with different feelings during the activities, supported by a social and emotional network made up of similar people, and learning to know each other, trust and tell their stories; learning new perspectives and meanings from themselves and from others; understanding their own story, reunifying past and present, thanks to an image of themself that becomes comprehensible to others and to those who tell it, seeing it with new eyes, in a different context. The group context allows them to experience the reality of the disease as something that can unite rather than isolate, something that can excite, make people laugh or cry together, restoring a sense of empowerment that was initially unthinkable [31].

Our team, together with the patients, identified the following common elements at the end of each group journey:

- Observation and familiarization with the “therapist’s communicative style” can foster the ability to understand the different languages and perspectives of reflection on the experience. Through attention and analysis of the patients’ history and comparison of connected experiences within the group, changes can be achieved in patients’ ability to give meaning to shared experiences and consequently to regulate the emotions related to them;
- starting from the experience of a relationship based on a contractualism or on an assent that appears to be the result of a decision that is still not very conscious, we note how, meeting after meeting, it takes on the connotation of free, conscious acceptance. Equality is therefore highlighted, which permeates the very meaning of group narrative encounters, both of power and responsibility [43];
- the births of new friendships are encouraged with the practice of sharing the experience in small groups. Sharing occurs by activating active, non-judgmental listening, an attitude of welcome and acceptance. The repetition of these experiences allows the participants to relive moments of sincere intimacy, alternating in the supportive role; this has the aim of promoting self-esteem and the feeling of being important and considered by others;
- the story of themselves, from the first meeting to the last, changes; participants can learn to know each other better, identify new strategies to deal with the difficulties of cancer, relate to themselves again, and regain possession of their lives;
- own the physical, psychic and spiritual-existential dimension and finally, learn to give meaning to suffering, to outline new life opportunities [44];
- the motivation and ability to begin to express parts of oneself that had not been expressed until then, due to socio-cultural fears regarding the disease, which after the group experience instead take on an identity trait, a factor of empowerment and genuine and creative adaptation [45];
- we move from relationships based on information to a dialectical-psychological-moral process, in which the relationship implies an interdependence between people, where trust and conscience meet [39]. What becomes most evident in the group narrative process is that the therapeutic aspect of the relationship is based primarily on values and dimensions that involve people [46].

On a psychic level, the use of the group path, the experience of existential equality, allows the emergence of new adaptive mechanisms, functional to a different maturity of the subject, which then appear to better respond to a new sense of self [47].

Factors such as intersubjectivity (being with others) and reciprocity (being for others) become primary principles of narrative sharing in the therapeutic group. We would like to quote Natoli in this sense (Manara DF, 2004) [48] when he recalls from an ethical-social perspective: “each of us exists by virtue of the others...”, if he had not been welcomed, loved, he would soon have left the world; none of us would be in the world if someone had not taken care of us, and taken responsibility for us. Responsibility is based on welcoming (Greek: offering hospitality). The group therapeutic path aims to allow “the awareness of moral friendship between equals, inscribed in the social common-union founded on the ontological dimension of finitude and on the ontic dimension of illness, to open up treatment relationships” [39].

## Data Availability

All data can be found on Google Scholar, university scientific research and Italian scientific conferenc.es.
